# Characterizations and antibacterial activities of passion fruit peel pectin/chitosan composite films incorporated *Piper betle L.* leaf extract for preservation of purple eggplants

**DOI:** 10.1016/j.heliyon.2022.e10096

**Published:** 2022-08-08

**Authors:** Thuy Thi Thanh Nguyen, Tu Quoc Le, Tuyet Thi Anh Nguyen, Lan Thi My Nguyen, Duyen Thi Cam Nguyen, Thuan Van Tran

**Affiliations:** a Faculty of Science, Nong Lam University Ho Chi Minh City, Ho Chi Minh City 700000, Viet Nam; b University of Science, Viet Nam National University Ho Chi Minh City, Ho Chi Minh City 700000, Viet Nam; c Institute of Applied Technology and Sustainable Development, Nguyen Tat Thanh University, 298-300A Nguyen Tat Thanh, District 4, Ho Chi Minh City 755414, Viet Nam; d NTT Hi-Tech Institute, Nguyen Tat Thanh University, 300A Nguyen Tat Thanh, District 4, Ho Chi Minh City 755414, Viet Nam

**Keywords:** Antibacterial activity, Biodegradable film, Food packaging, Pectin, Passion fruit peel

## Abstract

The present study aimed to synthesize biodegradable films based on crosslinked passion fruit peel pectin/chitosan (P/CH) films incorporated with a bioactive extract from *Piper betle L*. leaf, and investigate their morphological, mechanical, water vapor permeability, optical, and antibacterial properties. The thickness and water vapor permeability of P/CH blend films were proportional to the increasing concentration of *Piper betle* extract (PB). The tensile strength of P/CH/PB films was significantly reduced at 42.89% compared to the P/CH films. The morphological characterization affirmed that resultant blend films showed a well-organized homogeneous structure with no cracks. Moreover, the antibacterial activities against *Staphylococcus aureus, Pseudomonas aeruginosa*, *Bacillus cereus*, and *Klebsiella pneumoniae* increased with the increased concentration of PB in the obtained films. Our results demonstrated that P/CH/PB blend films could be potentially used for food packaging applications.

## Graphical abstract

**Figure:**
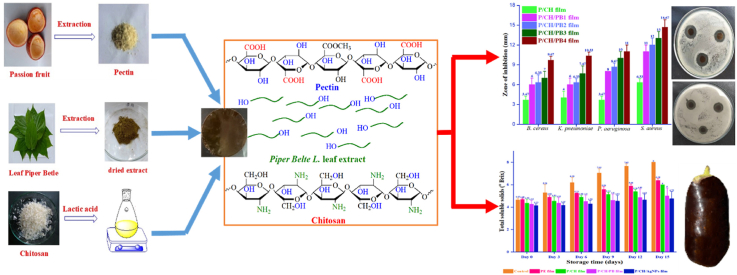

## 1 Introduction

The usage of biopolymers in packaged food, bottle packaging, and food coating is currently attracting huge attention due to their eco-friendliness, biodegradation, and biocompatibility. Among the representative biomaterials, polysaccharides (e.g., starch, cellulose, pectin, alginate, chitosan), proteins (e.g., soy protein, caseinates, corn zein, whey protein) and lipids (e.g., beeswax, oils, free fatty acids) have played an important role in the formation of innovative sustainable packaged food. Biopolymers have many emerging advantages, i.e., improving food safety, maintaining food quality, and extending food shelf life. Moreover, they reduce the generation and disposal of nondegradable plastic [1]. However, biopolymers still remain limited mechanical and physical properties, narrowing their potential in food packaging application. Searching for biopolymers with reinforced properties is therefore necessary to produce efficient packaging films for a number of specific purposes [2]. Among the polysaccharides, pectin is a complex polysaccharide containing D-galacturonic acid through the α-1,4-glycosidic bond, which exists in the primary cell walls or middle lamella of plant species [3]. The majority of commercial pectin can be directly extracted from citrus peel, apple fruit and other plants [4]. Pectin is also extracted from other sources such as soybean hull [5], mango peel [6], sunflower head [7], banana peel [8], passion fruit peel [9], durian rind [10], grapefruit peel [11], and persimmon peel [12]. Due to its high capacity of gel formation, pectin is used as stabilizer, emulsifier, gelling and thickening agent. Considering physicochemical properties, pectin is a biodegradable and biocompatible anionic nature polymer with a branched structure. It can be soluble in water and insoluble in organic solvents, and employed for film-forming polysaccharide [13].

In recent years, pectin has been considered as one of the main raw materials to make food packaging film due to its nontoxicity, edibility, natural abundance, low cost and selective gas permeability [14]. However, pure pectin films have many inherent drawbacks such as high water vapor permeability, and unsatisfactory mechanical features [15]. To settle these problems, blending pectin with other biopolymers such as cellulose, carboxymethyl cellulose, chitosan, alginate, starch, pullulan, agar, and poly (ethylene glycol) has been widely investigated [16, 17, 18, 19, 20]. Among typical candidates, chitosan is a hydrophobically cationic polysaccharide constituted by N-acetyl D-glucosamine and D-glucosamine units. It can be commercially obtained from the partial deacetylation of chitin [21]. This compound is widely applied in a variety of fields such as medicine, drug delivery, and food coatings owing to its biocompatibility, biodegradability, bioactivity, non-toxicity, low-cost, commercial availability, and excellent film-forming materials [22, 23]. Pure chitosan films possess good antimicrobial activities and mechanical properties as well as excellent permeabilities to gases (CO_2_ and O_2_) [24,25]. As a result, chitosan and pectin are compatible in the film matrix thanks to their electrostatic interactions and hydrogen bonding. Such incorporating protocol allows producing a composite membrane that holds key features of each component, enhancing their advantages towards mechanical, physicochemical, and permeable properties. In essence, chitosan is positively-charged NH_3_^+^ groups interacting with negatively-charged COO^–^ groups of pectin, leading to the formation of intermolecular in the film matrix. This interaction enhances the material properties of blended films as compared to those obtained from pure polymers alone [26]. In general, the properties of pectin-chitosan blending films can be affected by the nature of pectin (e.g., degree of esterification, mass molar and pectin source) [27] and chitosan (e.g. degree of deacetylation and molecular weight) [28] as well as the proportion of each component in the biopolymer blend film [29]. As a result, an investigation of molar ratio between pectin and chitosan is necessary.

Antimicrobial packaging is a type of active packaging containing antimicrobial agents that inhibit or prevent the growth of bacterial, but increase the shelf life of food products [30]. Moreover, antimicrobial packaging is proved to be more effective by a controlled migration of antimicrobial compounds into the food. Such key feature intercepts the undesirable microorganisms during food transportation and storage [31]. Interestingly, antimicrobial packaging can be effortlessly created by incorporating plant extracts into a polymer matrix or coating packaging films. These natural extracts contain a number of bioactive compounds which improve the antimicrobial properties of obtained films. For example, Han et al. [32] found kiwifruit peel extract in watermelon rind pectin (WRP) films, which significantly improved antioxidant activity, increased elongation at break, water vapor permeability, the opacity, and tensile strength of the films. In another study, pectin/chitosan films were loaded with tea-derived polyphenols, which were capable of inhibiting the chain reactions caused by free radicals, and hence reducing food damage [33]. Blended films of pectin and beetroot extract could be used as a pH indicator to monitor quality changes of chilled beef during storage [34]. Therefore, the use of such nature extracts for improving pectin/chitosan films has received much attention.

*Piper betle Linn.* leaf is known as a rich source of active phenolic compounds*. Piper betle L.* leaf belongs to the Piperaceae family, an important medicinal plant in South East Asia. *Piper betle* leaves were reported to contain a large number of biologically active components, such as chavicol, chavibetol, hydroxychaviol, piperbetol, piperol A, methylpiperbetol and piperol [35]. Additionally, the extract from *Piper betle* leaves has been studied for many biological activities including anti-inflammatory [36], anti-diabetic activity [37], anti-oxidant activity [38], anti-bacterial and anti-carcinogenic activity [39], and anti-histaminic activity [40]. Consequently, *Piper betle L.* leaf extract could be used as an additive to make bioactive food packaging films [41, 42]. To the best of our knowledge, this is the first work that reported the production of chitosan and pectin film derived from the passion fruit peel with the addition of *Piper betle L.* leaf extract. Therefore, the objective of the work was to produce passion fruit peel pectin-chitosan (PC) films containing *Piper betle L*. leaf extract, which was incorporated into PC films as a bioactive material. This study focused on investigating the physicochemical properties of the PC films containing *Piper betle L*. extract and determining their applicability as antimicrobial packaging film.

## 2 Experimental

### 2.1 Materials

All chemicals used for the experiment including citric acid (CAS No. 77-92-9, purity of 99.6%), ethanol (CAS No. 64-17-5, purity of 99.5%), glycerol (CAS No. 56-81-5, purity of 99.0%), lactic acid (CAS No. 50-21-5) were purchased from Acros. *Piper* betel leaf was collected from the local markets in Ho Chi Minh City, Vietnam. Distilled water was used in all experiments.

### 2.2 Microorganisms

The antibacterial activity of as-synthesized films could be measured using four different bacterial strains including Gram-positive (*Staphylococcus aureus* and *Bacillus cereus)*, and Gram-negative (*Pseudomonas aeruginosa* and *Klebsiella pneumoniae).* All the bacterial strains were obtained from the Department of Plant Biotechnology and Biotransformation, Faculty of Biology and Biotechnology, University of Science, Vietnam National University Ho Chi Minh City, Vietnam.

### 2.3 Preparation of composite packaging

#### 2.3.1 Passion fruit peel pectin extraction and characterization

Pectin was extracted from the passion fruit peel (PFP) using the acidic solvent. Addition of 4 g the dried PFP powder to 100 mL of 1 *M citric* acid solution at 85 °C with a magnetic stirrer for 90 min. After extraction, the mixture was filtrated through a filter cloth and then centrifuged at 5,000 rpm for 15 min. The aqueous extract was subsequently added in 96% ethanol and the solution was then kept under room temperature for 8 h. The precipitated pectin was collected and washed three times with 96% ethanol to remove impurities and dried in a hot air oven for 50 °C for 24 h. The dried pectin was stored at room temperature for the next experiments.

#### 2.3.2 Extraction of *Piper betle* L. Leaf extract

*Piper betle L.* leaves (PBL) were washed with tap water to remove some dirties, cut into small pieces, and dried in an oven for 24 h at 50 °C. The dried leaves were ground into powder by using a blender. 5 grams of dried powder were extracted with 150 mL ethanol using the Soxhlet apparatus at 78 °C for 2 h. The extract was then concentrated by using a rotary evaporator to eliminate the solvent and only get the dried extract. The crude extract was allowed to cool down at ambient temperature and kept the refrigerator at 4 °C for further use. Total phenolic compounds of PBL were determined through the methodology described by Ribeio et al. [43].

#### 2.3.3 Preparation of pectin/chitosan/PB films

**Figure 1:**
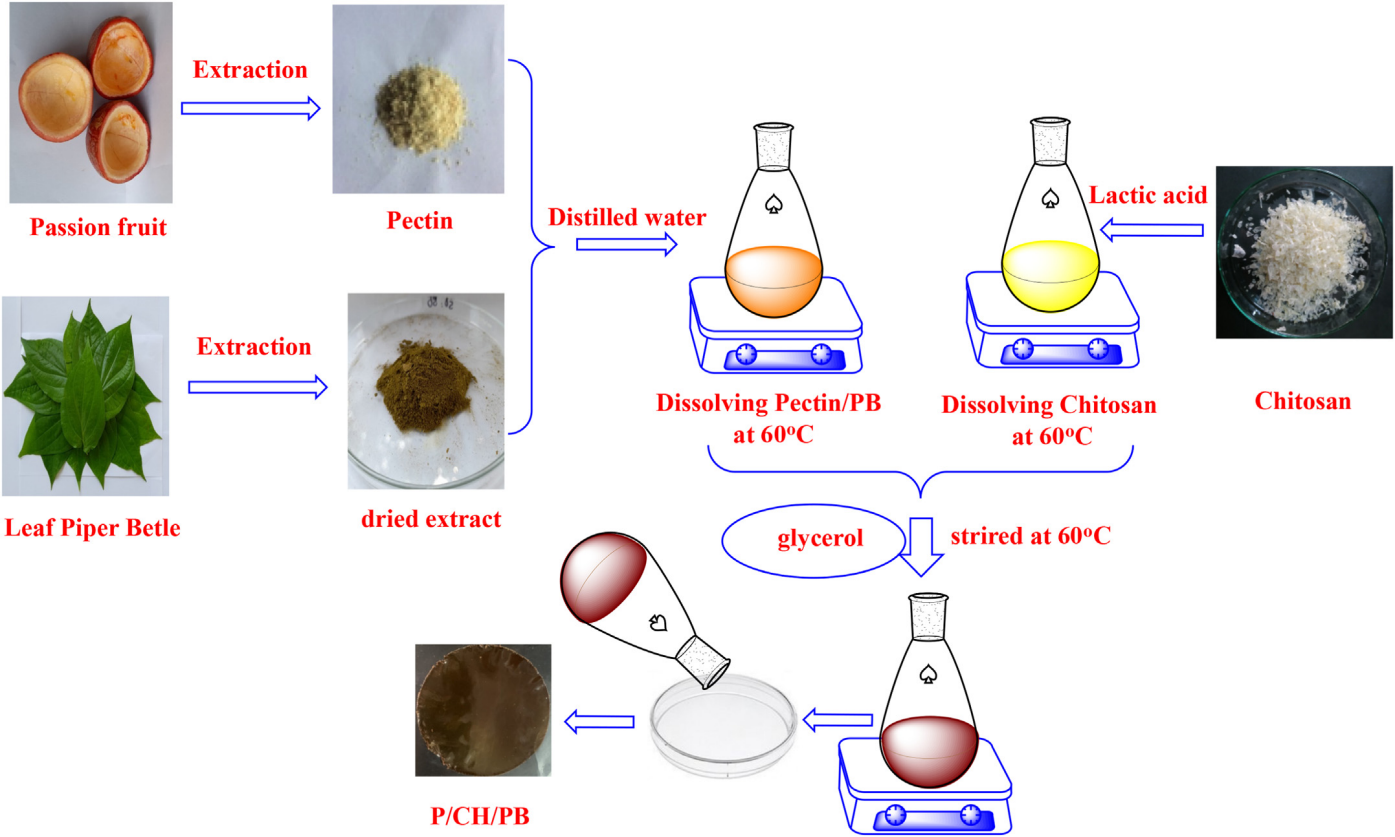
The descriptive preparation process of pectin/chitosan/PB films.

Pectin/chitosan/PB films were prepared following the methodology previously described by Murek et al. [44] with some modifications as shown in Figure 1. Briefly, the crude extract of leaf *Piper betle* was added in pectin solution (2%, w/v) to reach the final concentrations of 0%, 0.5%, 1.0%, 1.5%, and 2.0% (w/w, based on the weight of pectin in mixture). Then, the mixture was gradually added into chitosan solution (2%, w/v, 2% of lactic acid solution used as solvent) with continuous stirring to reach the final ratio of pectin and chitosan at 1:1 (w/w). Glycerol as plasticizer (30% in respect to the weight of the polymer in the mixture, w/w). Afterward, blends were heated at 60 °C under magnetic stirring for 120 min and put in the Petri dishes with a radius of 10 cm (30 mL mixture per dish). Subsequently, all dishes were dried in an oven at 50 °C for 48 h and the solid films were then peeled off from the dishes and kept in a desiccator for further studies. The films produced were codified as P/CH/PB0; P/CH/PB1; P/CH/PB2; P/CH/PB3; P/CH/PB4.

### 2.4 Characterizations of the films

#### 2.4.1 Film thickness

Films thickness was measured at five random sites using a micrometer (QLR digital-ỊP4, China) with 0.001 mm accuracy and average thickness film was expressed in millimeters (mm).

#### 2.4.2 Water solubility (WS)

Before the test, the P/CH film and P/CH films with different concentrations of PB leaf extract were dried in an oven at 105 °C for 24 h. To determine water solubility, the films were cut into 4 cm × 4 cm pieces and then weighted to obtain the initial dry mass (W_dry_). After, each sample was immersed in a beaker containing 30 mL of distilled water and incubated at room temperature for 24 h. After, films were withdrawn from the water and dried at 105 °C for 24 h. The water solubility of the films was defined by the following equation (Eq. 1).

(1) $$\text{Water}\;\text{solubility}\;\left(\%\right)=\frac{W_{i}-W_{f}}{W_{i}}\times 100$$

where W_i_ is the initial weight (g) of dry film, W_f_ is the final weight (g) of the film after the drying process. All measurements were done in triplicate.

#### 2.4.3 Water vapor permeability (WVP)

WVP was conducted to identify the ability of the P/CH films with and without the PB leaf extract to prevent water vapors from passing through the film. WVP was carried out according to the ASTM method with some modifications. The Erlenmeyer flasks were filled with 30 mL water. Then, the films were placed on the mouth of the Erlenmeyer flask and glued with wax. After being glued, the Erlenmeyer flasks and films were weighed (W_1_) and located in an incubator. The RH and temperature conditions of the Erlenmeyer flasks were controlled at 50 ± 5% and 27 ± 3 °C. The Erlenmeyer flasks and films were weighed every 24 h for 5 days (W_2_), the WVP (g mm h^−1^ m^−2^ kPa^−1^) was calculated by the following equation (Eq. 2).

(2) $$\text{WVP}=\frac{\left(W_{1}-W_{2}\right)\times a}{A\times h\times \Delta P}$$

where, a and A were the film thickness (mm) and surface area (m^2^) of the circular film, respectively; h and ΔP were the time (h) and vapor pressure difference (kPa) across the two sides of the film, respectively. The tests of WVP were performed in triplicate.

#### 2.4.4 Optical properties

The transparency of the obtained films was determined according to the method of Rincón et al [45]. The films were cut in rectangular strips (1.3 cm × 3.5 cm) and tested using a UV-vis spectrophotometer (Shimadzu UV-160A) at wavelengths between 200 and 800 nm. Each film was measured in triplicate and the average of three spectra was calculated. The transparency at 660 nm (T_660_) was obtained by the following equation (Eq. (3).

(3) $$\text{Transparency}\;\left(\%\right)=\frac{\log\left(\%T_{660}\right)}{a}$$

where, %T_660_ was the percent transmittance at 660 nm, and *a* was the film thickness (mm).

#### 2.4.5 Mechanical properties

Mechanical properties of the films were measured with Texture Analyzer TA-HD plus, following the standard ASTM D882. Film specimens with a dimension of 10 mm × 60 mm were cut from the obtained films and fixed between grips of the instrument, stretched at a rate of 20 mm/min until breaking. Elongation at break (% E) and maximum force were determined from force versus deformation curves. Tensile strength (TS) was obtained by dividing the maximum force by film cross-section. Young’s Modulus (YM) was calculated from the initial portion of the stress-strain curve. All the measurements were carried out at least five replicates.

#### 2.4.6 FT-IR analysis

The FT-IR spectra of the prepared films were recorded using FT-IR spectroscopy (Bruker-Tensor II, Bruker Optic GmbH, Karlsruhe, Germany). The measurements were performed at room temperature and spectra were recorded at wave numbers ranging from 400–4000 cm^−1^ with a step of 4 cm^−1^. This analysis aimed to determine the modifications induced by the incorporation of *Piper betle L.* leaf extract into the pectin/chitosan matrix at a molecular scale.

#### 2.4.7 X-ray diffraction (XRD)

XRD of the films was measured by an X-ray diffractometer (Shimadzu XRD-6000, Japan) with a scanning rate of 4°/min in the diffraction angle 2θ = 2°–80° using Cu-Kα radiation (λ = 1.54 Å) operated at 40 kV and 100 mA. The results were analyzed using Origin software.

#### 2.4.8 Scanning electron microscopy (SEM)

Films morphology was obtained using a scanning electron microscope (S-4800, Hitachi, Japan). The films were decorated with a platinum layer under the accelerating voltage of 10 kV.

### 2.5 Antibacterial activity test

Antibacterial activity of pectin/chitosan incorporated with *Piper betle L.* leaf extract was conducted against Gram-positive bacteria (*Staphylococcus aureus, Bacillus cereus)*, and Gram-negative bacteria (*Pseudomonas aeruginosa*, *Klebsiella pneumoniae*). *Staphylococcus aureus* was initially cultured in a brain-heart medium, while others were cultured in 5 mL of lysogeny broth (LB) medium for 12 h. Each assay was obtained from 100 μL of cultured bacteria (10^6^–10^7^ CFU mL^−1^). The samples were poured into culturing Petri plates. Afterward, an amount of agar was added into 3 wells, 8 mm in diameter for each. P/CH, P/CH/PB1, P/CH/PB2, P/CH/PB3, and P/CH/PB4 films were sliced to load in each well. Each experiment was repeated three times at 37 °C for 24 h. The size of the inhibition zone was used as an indicator to assess the antibacterial activity of films.

### 2.6 Application in situ of films in purple eggplant

The obtained films were applied to purple eggplant, to evaluate the postharvest conservation. To perform this, purple eggplant was packaged with the PE film, P/CH film and P/CH/PB film, in triplicate, and purple eggplant without packaged was used as the control in the experiment. Total soluble solids (°Brix) were measured by direct reading of a double scale Incoterm portable refractometer during 15 days of storage at room temperature.

### 2.7 Statistical analysis

The experimental data were analysed using the SAS software (SAS Institute. Inc., Cary, NC, USA). All experiments were carried out at least three times, and the data were represented as mean ± standard deviation.

## 3 Results and discussion

### 3.1 Passion fruit peel pectin extraction and characterization

The chemical characteristics and extracted yield of passion fruit peel pectin were displayed in Table 1. The yield of the pectin extraction in this study was 16.16 ± 0.05 with an average molar mass of 20.81 kDa, close to those of Liew et al. (14.60%) [46], Seixas et al. (18.2%) [47] under different extraction conditions, using tartaric acid as an extractor. The degree of esterification (DE) and galacturonic acid (GA) content were 50.74 ± 0.71% (high methoxyl pectin) and 62.92 ± 0.02%, respectively, similar to that obtained by de Oliveira et al. [48]. The polydispersity index (Mw/Mn) of the isolated pectin was 2.15, indicating a broader range of molecular weight distribution in pectin macromolecule since this polymer was obtained from a natural source [43]. Moreover, the total phenolic compounds of *Piper betle L.* leaf extract was determined at 64.703 ± 1.963 mg/g. This finding indicates that *Piper betle L.* leaf extract can contain a proper amount of antioxidant, which was helpful to the application of P/CH films. Thus, pectin obtained from passion fruit peel could be used as a polymeric matrix to prepare food packaging films.

**Table 1:** The chemical characteristics of passion fruit peel pectin.

Yield (% by w/w)	Ash (%)	GA (% by w/w)	DE (%)	Mn (kDa)	Mw (kDa)	PDI
16.16 ± 0.05	1.01 ± 0.11	62.92 ± 0.02	50.74 ± 0.71	20.81	44.64	2.15

### 3.2 Physical properties of films

#### 3.2.1 Visual aspect, thickness, water vapor permeability (WVP), and transparency

**Figure 2:**
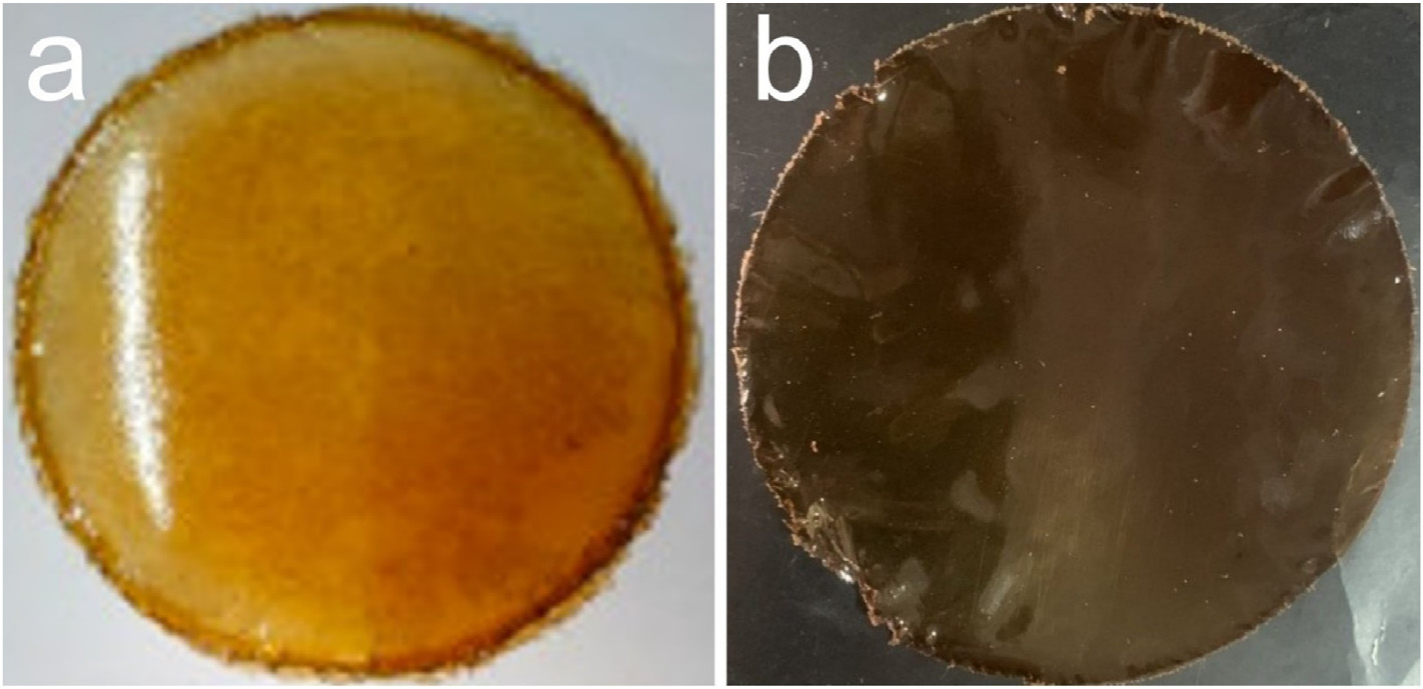
The photographs of the P/CH films without (a) and with 2.0% PB extract (b).

The digital photos of the P/CH films with and without the PB extract were shown in Figure 2. The films displayed odorless, flexibility and absence of any cracks when remove off the Petri plates. It could be seen that the P/CH after the addition of PB extract turned brown.

The thickness, WVP, and optical (transparency) values obtained for the P/CH films with different concentrations of PB extract were displayed in Table 2. As shown, the addition of PB extract increased the thickness of films, which followed by the following order: 0.177, 0.185, 0.195 and 0.213 mm for P/CH (0.100 mm) < P/CH/PB1 (0.177 mm) < P/CH/PB2 (0.185 mm) < P/CH/PB3 (0.195 mm) < P/CH/PB4 (0.213 mm). This trend can be due to the increased supplementation of soluble solids from PB extract into the film matrix. Similar results were observed by Filipini et al. [49], who undertook incorporation of jambolão skin extracts in the methylcellulose films. Santos et al. [50] also revealed the similar observation of adding purple onion peel extract into functionalized sodium alginate active films. Accordingly, the sodium alginate films incorporated with the purple onion peel extracts significantly increased the values of film thickness (0.049–0.062 mm). In contrast to the above works, Luo et al. [51] observed a decrease in the thickness of the sodium alginate film containing guava leaf water extract.

**Table 2:** Thickness, water vapor permeability, and transparency of P/CH films with different concentration of *PB* leaf extract.

Films	Thickness (mm)	WVP (g·mm/h·m^2^·kPa)	Transparency (%)
P/CH	0.100 ± 0.025	1.20 ± 0.07	19.33 ± 0.61
P/CH/PB1	0.177 ± 0.031	1.34 ± 0.04	10.48 ± 0.11
P/CH/PB2	0.185 ± 0.006	1.37 ± 0.02	10.19 ± 0.17
P/CH/PB3	0.195 ± 0.010	1.56 ± 0.05	9.63 ± 0.56
P/CH/PB4	0.213 ± 0.015	1.77 ± 0.01	8.52 ± 0.59

The WVP is a crucial parameter indicating the barrier capacity of packaging materials to water vapors. The WVP values of P/CH films as a function of the PB extract contents were also shown in Table 2. The WVP values of P/CH films incorporating with the PB extract were higher compared with that of the control film. It was observed that the concentration of the PB extract in the P/CH films increased, the WVP increased. These results can be explained as the addition of PB extract into P/CH matrix increased the concentration of polar groups which caused to increase the availability of free hydroxyl groups in the matrix to react with water hence the moisture sensitivity of the films was increased. Torres-León et al. [52] suggested that the water vapour permeability of the P/CH/PB films was increased due to the relatively loss of intermolecular interaction of the polymer, increasing the free volume of the film matrix, which led to the water molecules passing into the film. The addition of *Caesalpinia ferrea Marties* extract also increased the WVP of PVA film [53]. Shufang et al. [54] reported the same increase in the WVP on gum arabic/cellulose nanocrystal – biofilms added with grape skin extract, pomegranate peel extract, and red pitaya peel extract. The effects of the concentration of the PB extract on the transparency of the P/CH films were displayed in Table 2. When the PB extracts were incorporated into P/CH films, the decrease in transparency of the film was inversely proportional to the increase in concentration of PB leaf extract added into the film matrix. Specifically, the transparency of P/CH film with 2.0% of PB leaf extract was the lowest value (8.52 ± 0.59%) and the transparency of the film control was the highest (19.33 ± 0.61%). We assumed that the presence of phenolic compounds in the PB leaf extract may affect the transparency of the films. Less transparency of films could be advantageous for food protection by reducing the direct emission of light on these products [55]. Therefore, the incorporation of plant extracts into films enhanced the light barrier capacity of food packaging.

#### 3.2.2 Mechanical properties

The mechanical properties are important indexes of composite packaging membranes [56]. To evaluate the effect of PB leaf extract on the mechanical properties of the films, the tensile strength, elongation at break, and elastic modulus were measured as shown in Table 3. It was found that the PB leaf extract incorporation into the P/CH films led to a decrease in the tensile strength from 16.91 to 10.09 Mpa. Specifically, the P/CH films incorporated with PB leaf extract had a reduction of up to 42.89% of tensile strength as compared to the control film. The decrease in tensile strength may be because the PB leaf extract filled in the gap between pectin and chitosan, weakening the interactions of these biopolymers, increasing the free volume and mobility of polymer chains inside the film matrix [57]. Moreover, it was observed for a significant decrease in the elongation at break of P/CH/PB films in contrast with control P/CH film. This decrease might be attributable to the bonding interruption of polymeric networks in the presence of *Piper betle* leaf extract. We assume that some bioactive compounds such as polyphenols in the extract change the physicochemical properties of the membrane, leading to a breakage of polymeric network. Our findings well agreed with Moreno et al. [58], who worked with agar, alginate, and agar/aginate based films with *Larrea* nitida extract. Ju et al. [59] also reported the same changes in mechanical properties in furonan-based film incorporated with yellow onion peel extract.

**Table 3:** Mechanical properties of P/CH film with different concentrations of PB leaf extract.

Films	Tensile strength (MPa)	Elongation at break (%)	Elastic modulus (MPa)
P/CH	17.67 ± 1.50	23.27 ± 4.34	450.09 ± 222.97
P/CH/PB1	16.91 ± 4.65	16.06 ± 10.19	366.22 ± 134.62
P/CH/PB2	14.32 ± 0.69	22.24 ± 4.33	221.40 ± 10.90
P/CH/PB3	12.32 ± 2.41	28.03 ± 6.28	205.76 ± 70.44
P/CH/PB4	10.09 ± 0.97	20.33 ± 4.83	200.50 ± 79.80

#### 3.2.3 FT-IR spectroscopy analysis

**Figure 3:**
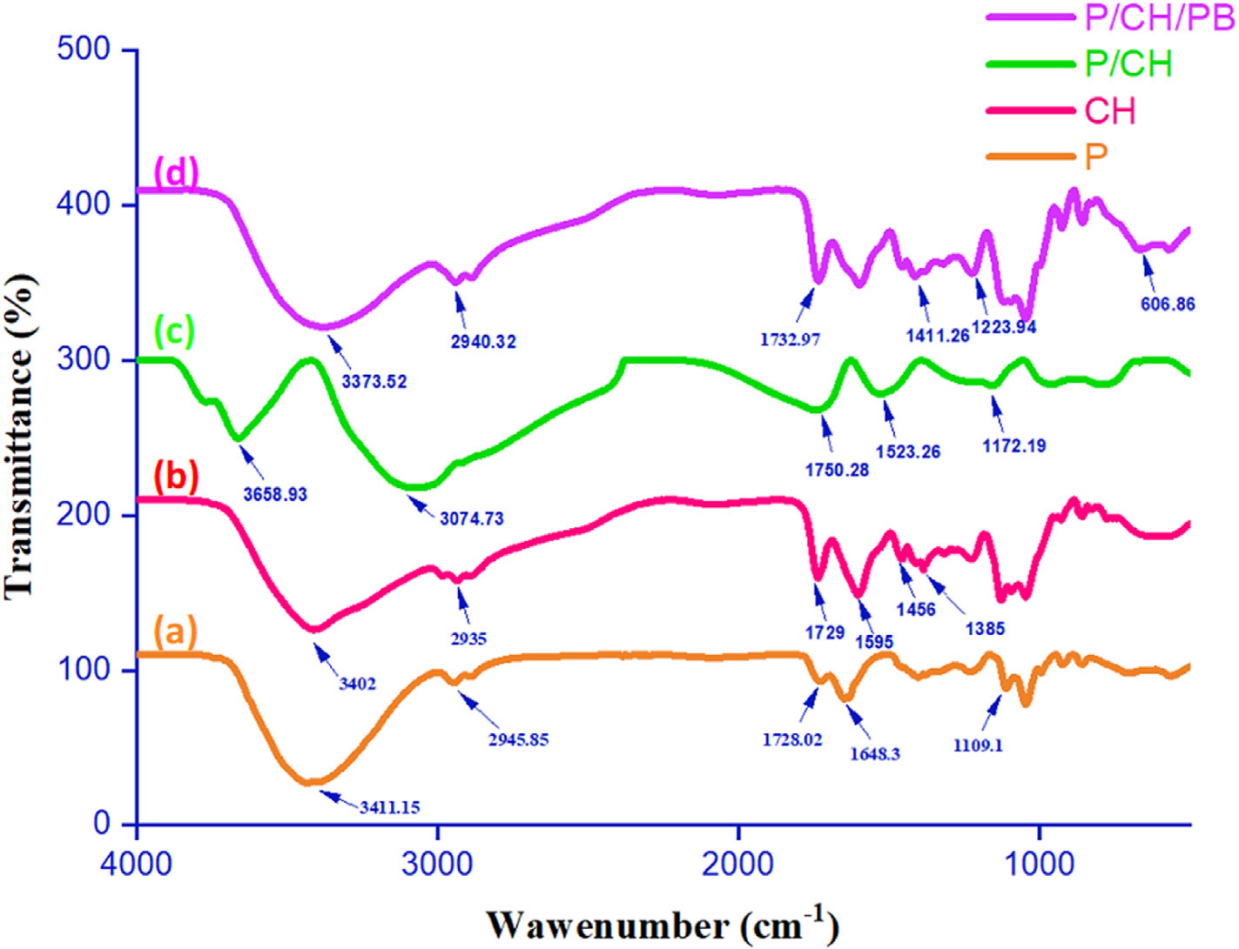
FT-IR spectra of passion fruit pectin films (a); chitosan films (b); passion fruit pectin/chitosan films (c); passion fruit pectin/chitosan/*Piper betle* leaf extract films (d).

**Figure 4:**
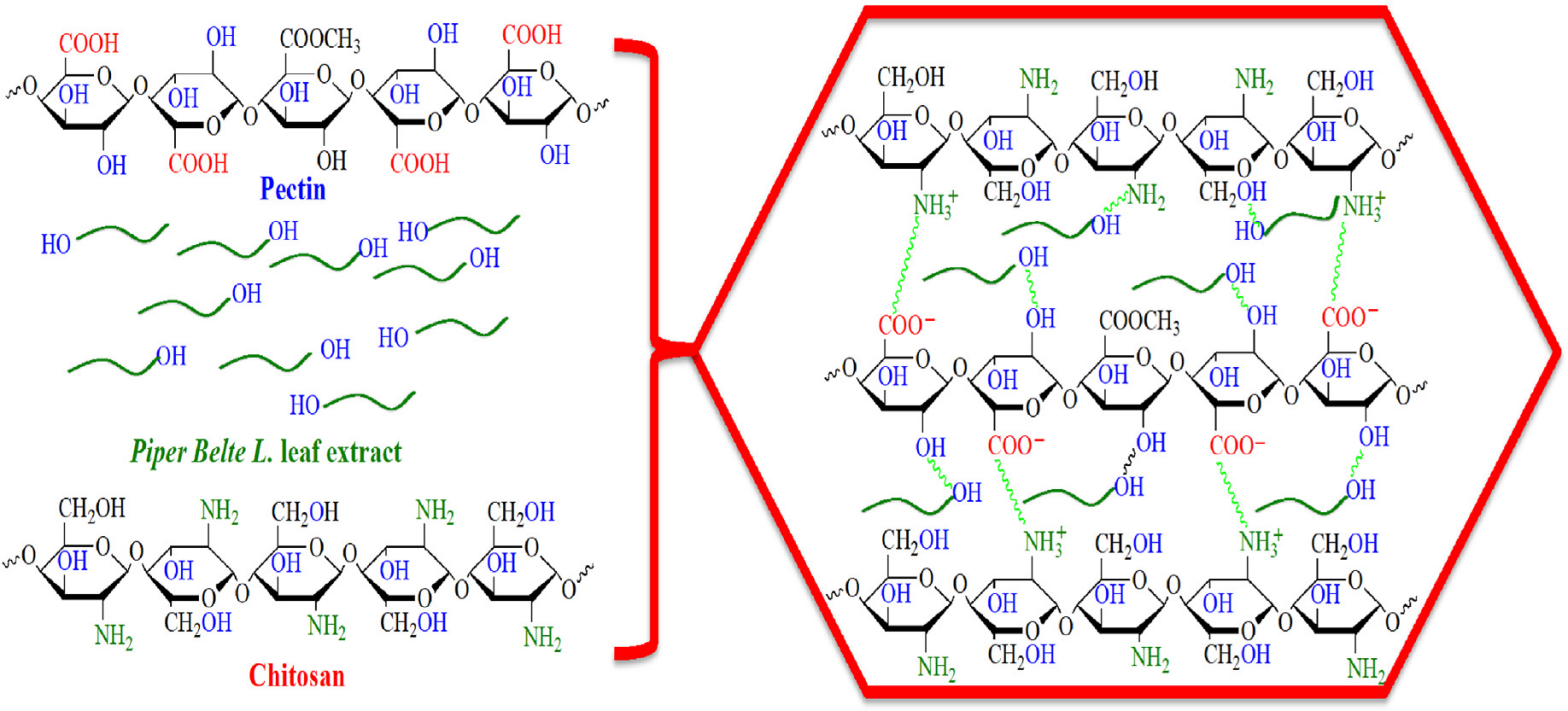
Schematic illustration of the chemical interactions between phenolic compounds in *Piper betle* leaf extract and functional group of pectin and chitosan.

FT-IR analysis was carried out to understand the chemical interactions between functional groups of passion fruit pectin, chitosan, and those of *Piper betle* leaf extract in the film matrix. The FT-IR of passion fruit pectin, chitosan, passion fruit pectin/chitosan, and passion fruit pectin/chitosan/*Piper betle* leaf extract films are shown in Figure 3. For the passion fruit pectin film (Figure 3a) the broad band at 3411 cm^−1^ is attributable to the stretching vibrations of hydroxyl groups O–H of pectin [60]. The band at 2945 cm^−1^ was ascribed to C–H vibrations of methylene group. The band at a wavelength of 1728 cm^−1^ corresponded to C–O and C=O bonds of esters. Bands at 1648 cm^−1^ and 1401 cm^−1^ could be associated with the symmetric and asymmetric stretching of carboxylic groups, respectively. Another summit at 1230 cm^−1^ was ascribed to C–O vibrations of carboxylic acids. In addition, other footprints at 1109 cm^−1^ and 1044 cm^−1^ might be attributable to C–O–*C stretchings* of saccharide compounds [61]. In the case of chitosan film (Figure 3b), the peaks situated at 3402 cm^−1^ and 2935 cm^−1^ were attributed to the O–H group of chitosan and lactic acid molecules in the film. An absorption band at 2935 cm^−1^ corresponding to C–H stretching was observed. Also, the characteristic absorption bands at 1595 and 1385 cm^−1^ were assigned to N–H bonds of primary amide and tertiary amide, respectively [62, 63]. The peak at 1456 cm^−1^ was called for bending vibrations of hydroxyl groups. Besides, an absorption band at around 1729 cm^−1^ was possibly attributed to the stretching of carboxylic groups of lactic acid. The spectrum of the passion fruit pectin/chitosan blend films (Figure 3c) showed that major shifts ranged from 1800 to 1500 cm^−1^, which demonstrated the interaction between amino and carboxyl groups. In fact, a footprint at 1648 cm^−1^ is characteristic of pectin in harmony with a symmetrical carboxylic stretching. A typical band at 1595 cm^−1^ for chitosan corresponded to the bending vibration of N–H plane of primary amide were changed to the peaks at 1792 cm^−1^ and 1523 cm^−1^, indicating an interaction between the COO^–^ groups of pectin with the NH_3_^+^ groups of chitosan, which affirms the creation of intermolecular ionic salt bonds between pectin and chitosan chains. The FT-IR spectra of the passion fruit pectin/chitosan blend films with *Piper* betel leaf extract exhibited some appreciable changes compared to the control film (Figure 3d). Accordingly, absorption peaks at 3373 cm^−1^, 2940 cm^−1^, 1729 cm^−1^, and 1597 cm^−1^, which were observed on the pectin/chitosan/piper betel leaf extract films shifted to lower wavenumbers at 1455 cm^−1^, 1114 cm^−1^, and 1090–1030 cm^−1^ for the blend films. Such phenomenon possibly indicated the presence of C–H bending vibrations of alkane, C–C stretching vibrations in aromatic and aliphatic amines of *Piper* betel leaf extract and C–O–C stretching, respectively [64]. Furthermore, a new absorption at 1411 cm^−1^ arose, which could be due to the electrostatic interactions and hydrogen bonds between phenolic compounds in *Piper betel* leaf extract and pectin/chitosan films (Figure 4). It has been reported that the original chemical bond constant of polysaccharides might be decreased by the strong hydrogen bond between hydroxyl groups of pectin or amino groups of chitosan and hydroxyl groups of phenolic compounds in PB extract [65]. Consequently, absorption frequency leans towards the low wavenumber. Han et al. [33] showed the same outcomes as loading tea polyphenol into the pectin and chitosan matrix.

#### 3.2.4 XRD analysis

**Figure 5:**
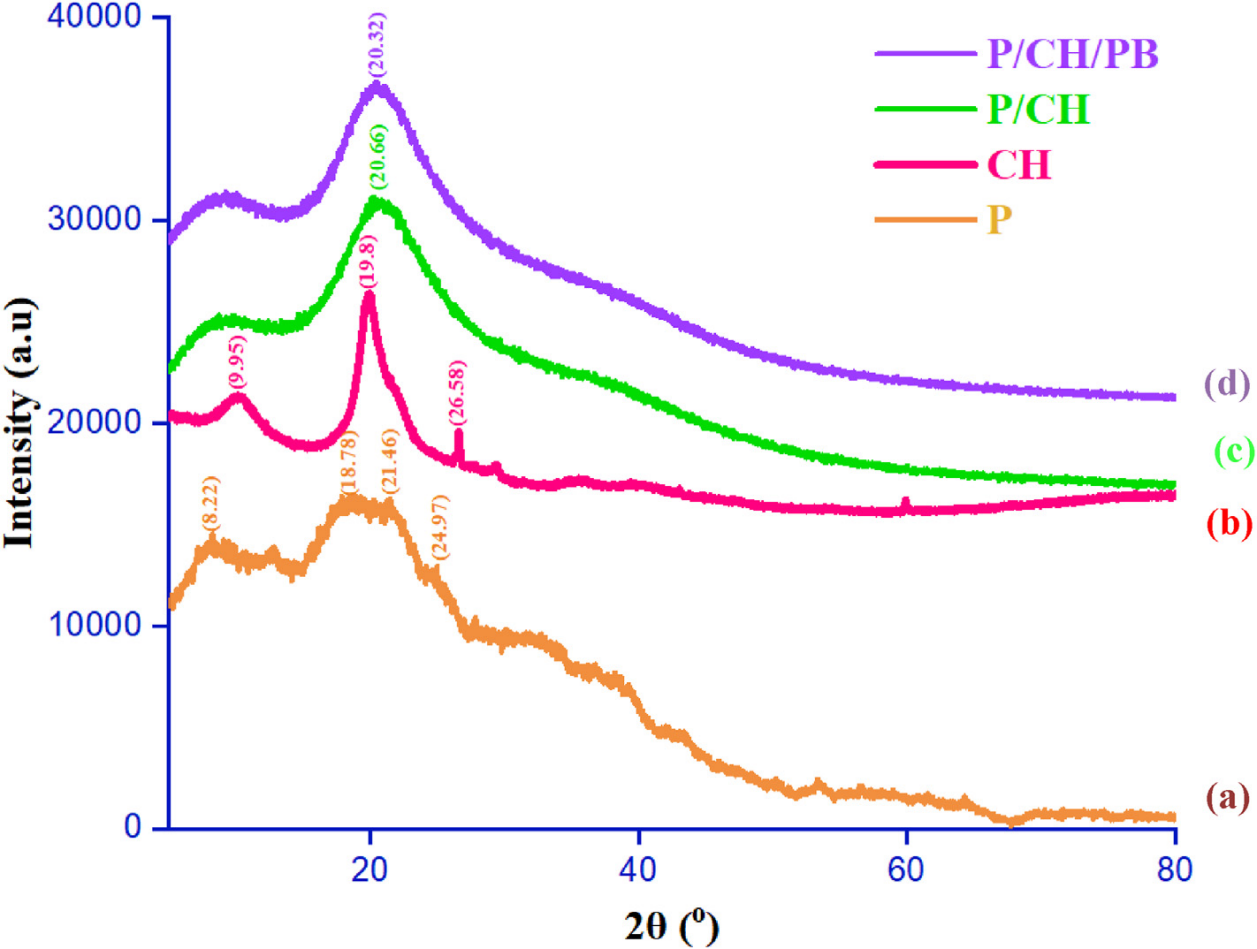
XRD diffraction patterns of pectin film (a), CH film (b), P/CH film (c), and P/CH/PB film (d).

The XRD analysis was carried out to explore the crystalline structures possibly presented in the film components. The patterns of P, P/CH, and P/CH/PB films are depicted in Figure 5. For P film, a broad crystalline peak was observed at 2θ = 15–30°, indicating that the amorphous nature of pectin. This result was in harmony with a previous work by Aditi et al. [66], therefore, the pectin has been successfully produced. Obviously, the XRD pattern for the blend without PB leaf extract showed the crystallinity due to a typical peak at 20.66°. Such patterns represent that the pectin/chitosan films were quasi-amorphous form and weakly crystallized, possibly related to the semi-crystalline nature in chitosan structure. This indicated that the strong interactions such as hydrogen bond and ionic interaction between pectin and chitosan in the matrix of the blend films may be deconstructed, causing the close packing of chitosan molecules to form the regular crystalline [67]. Incorporation of PB leaf extract into P/CH blend films insignificantly changed the peak position (20.32°) in the XRD pattern. Nevertheless, the peak intensity in the XRD spectra of the P/CH/PB slightly diminished in comparison with the P/CH films. We suggested that the PB leaf extract was molecularly dispersed into biopolymer matrix, creating some interactions of phenolic compounds of the PB leaf extract with the pectin and functional groups of chitosan. It could be, therefore, concluded that the incorporation between PB extract with biopolymer matrix did not affect the amorphous structure of the blend films.

#### 3.2.5 Morphology

**Figure 6:**
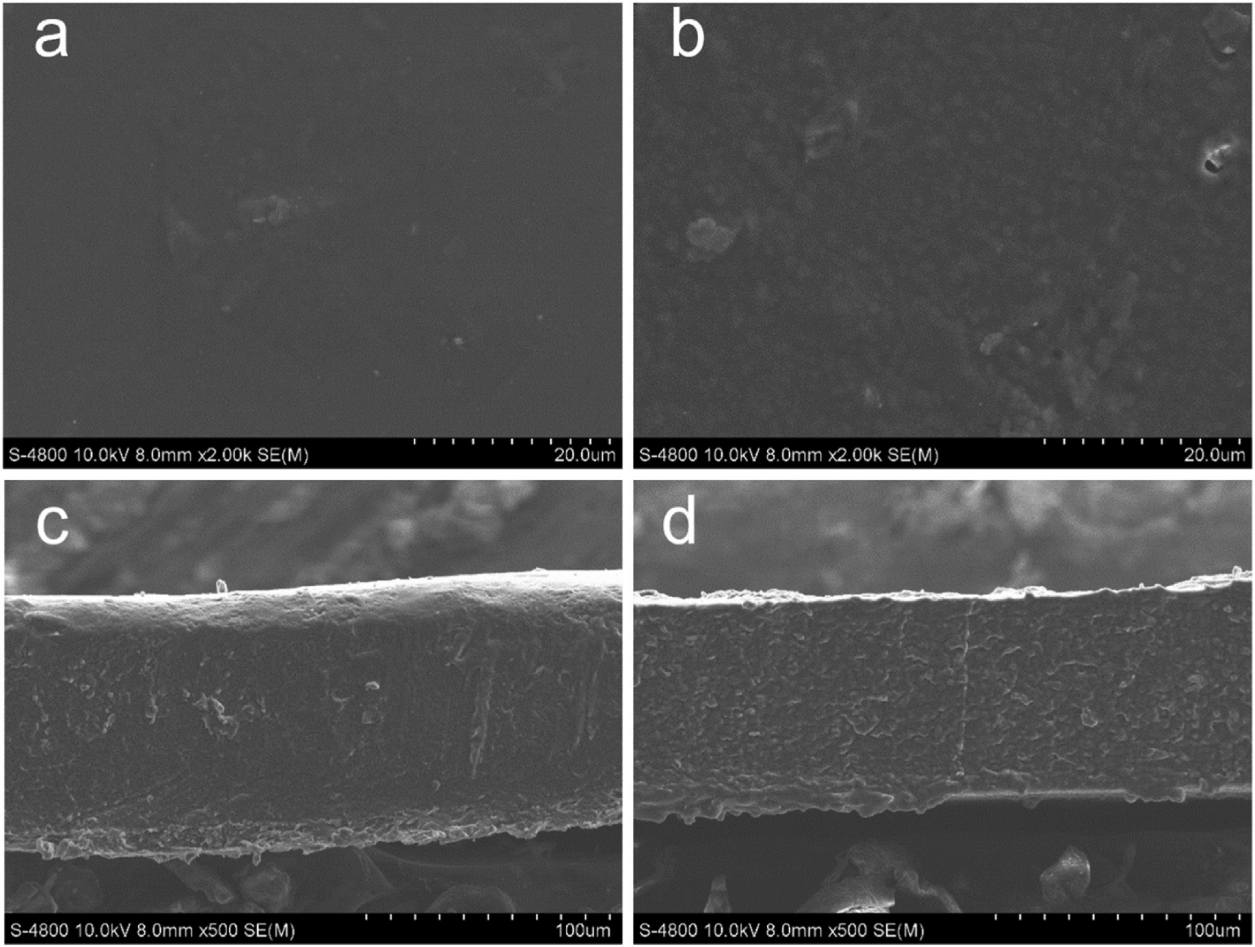
SEM micrographs of P/CH film containing PB leaf extract. Surface morphology: (a) P/CH film, and (b) P/CH/PB film. Cross-section: (c) P/CH film, and (d) P/CH/PB film.

The microstructure of film depends on the interactions between the material compositions, which influence the final properties of the films such as thickness, optical, mechanical, and barrier and antibacterial properties [68]. The surface and cross-section morphology of the P/CH and the film with PB leaf extract were shown in Fig. 6a, b. The surface of the P/CH film was homogeneous, regular without any noticeable pores or cracks, demonstrating high compatibility between chitosan and pectin to form a blend. After adding the PB leaf extract into the film matrix, the film surface showed relatively smooth with several pores that related to the partial release of bioactive compounds in the leaf extract such as alkaloids, phenolic acid, amino acid on the film surface [69]. Han et al. [70] reported the same result in Noni fruit polysaccharide films incorporated with blueberry leaf extract. It is noticeable that the cross-section structure morphology of the P/CH film and the P/CH/PB film had some differences. The cross-section of P/CH film (Figure 6c) displayed more compact and homogeneous microstructure without any particular features, indicating excellent compatibility between pectin and chitosan. With the addition of the PB leaf extract into the film-forming matrix, however, the cross-section of the P/CH/PB film (Figure 6d) showed the dense structure with small pores and cavities. There might be a strong interaction between the plant extract and the biopolymer components. Other types of films also changed in their morphology after the addition of the plant extracts such as chitosan films with carvacrol and grape seed extract [71], fish gelatin film incorporated mango peels extract [72].

### 3.3 Antibacterial properties of films

**Figure 7:**
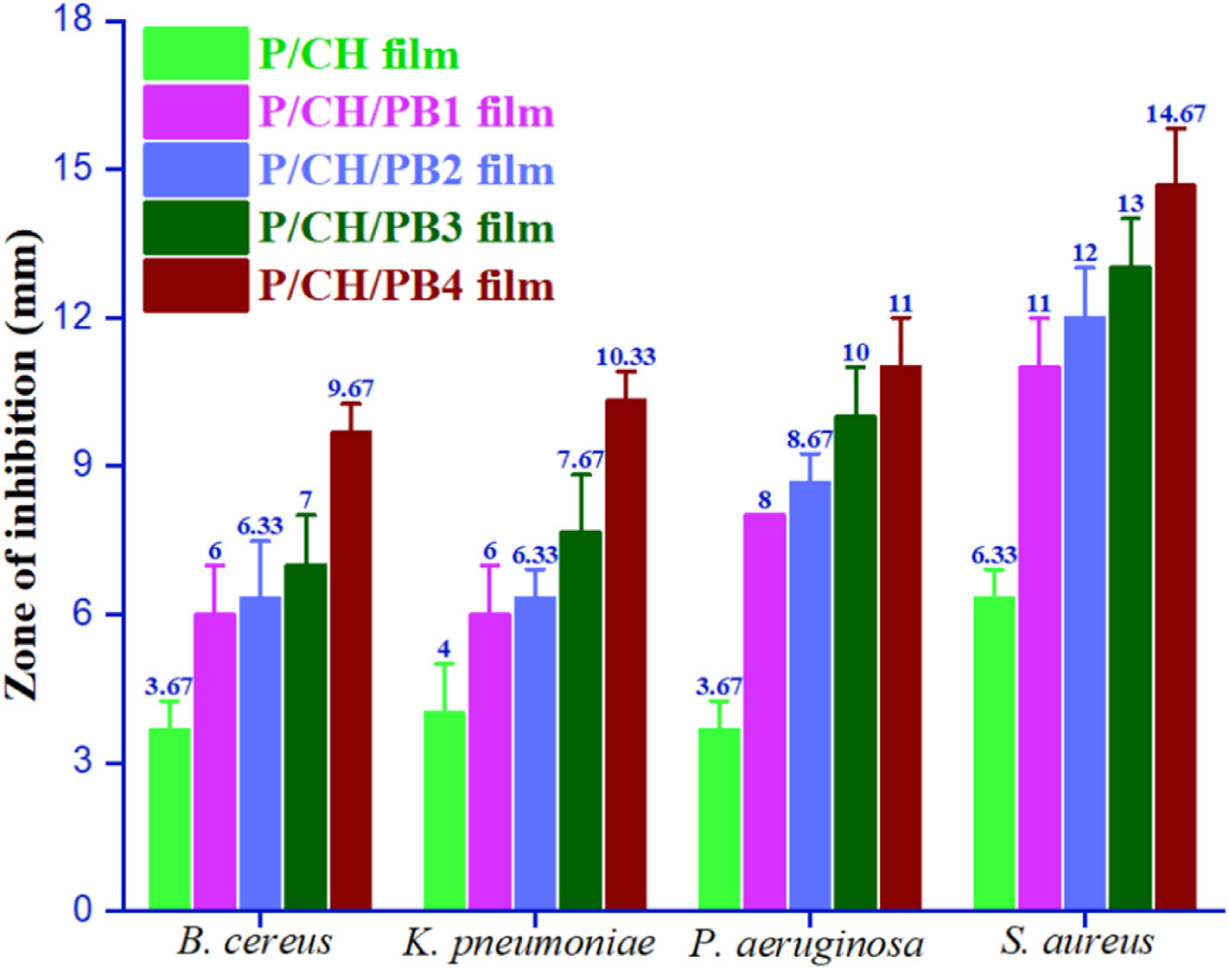
Antibacterial activities against pathogenic bacteria of P/CH film (neon green color), P/CH/PB1film (lavender color), P/CH/PB2 film (blue color), P/CH/PB3 film (forest color) and P/CH/PB4 film (brick color).

The antimicrobial activities of films were investigated against a panel of bacterial strains, namely *Staphylococcus aureus, Bacillus cereus*, *Pseudomonas aeruginosa*, and *Klebsiella pneumoniae* using the disk diffusion method. The visual zones of inhibition of films were recorded after 24 h exposure as presented in Table 4. All P/CH/PB films were found to be more effective against both Gram-positive and Gram-negative bacterial than the control film. In fact, the P/CH/PB films containing 0.0% (P/CH), 0.5% (P/CH/PB1), 1.0% (P/CH/PB2), 1.5% (P/CH/PB3), and 2.0% (P/CH/PB4) of PB leaf extract presented the larger zones of inhibition against *Staphylococcus aureus* at 6.33 ± 0.18, 11.00 ± 0.31 mm, 12.00 ± 0.15 mm, 13.00 ± 0.14 mm and 14.67 ± 0.26 mm, respectively (Figure 7). This result indicated that incorporation of the PB leaf extract and the biopolymer components increased the antimicrobial activity of P/CH/PB films. In addition, the increase in the antimicrobial activity was proportional to the concentration of PB leaf extract used in the film formulation. Indeed, the P/CH containing 2.0% of PB leaf extract in its composition, displayed the highest antimicrobial activity compared to those with 0.5%, 1%, and 1.5% of PB leaf extract. This could be explained by a range of phytochemicals in the PB leaf extract including carbohydrates, tri-terpenoids, steroids, alkaloids, eugenol, phytol, amino acid, and tannins acted as antibacterial agents [73]. The zone of inhibition of the Gram-negative *P. aeruginosa* bacteria, on the other hand, was found to be lower than of *S. aureus*, possibly due to the difference between the structures of the outer cell membranes of these microorganisms, which have a thinner outer lipopolysaccharide layer in their membrane, limiting the diffusion of hydrophobic compounds [74]. Dan et al. [75] also observed the same antimicrobial activity of chitosan-gelatin film incorporated with the extract from hop plant was more effective against Gram-positive than against Gram-negative bacteria. Thus, the natural compounds from PB leaf extract and incorporated in the biopolymer matrix might be excellent alternatives for food packaging materials. The obtained results suggest that the P/CH/PB films can be applied as an antibacterial active food packaging to prolong the shelf life of food.

**Table 4:** Antibacterial activities against pathogenic bacteria of P/CH film, P/CH/PB1 film, P/CH/PB2 film, P/CH/PB3 film and P/CH/PB4 film.

Films	Bacterial strains			
	*Pseudomonas aeruginosa*	*Staphylococcus aureus*	*Klebsiella pneumoniae*	*Bacillus cereus*
P/CH				
P/CH/PB1				
P/CH/PB2				
P/CH/PB3				
P/CH/PB4

### 3.4 Application of films as purple eggplant conservation

**Figure 8:**
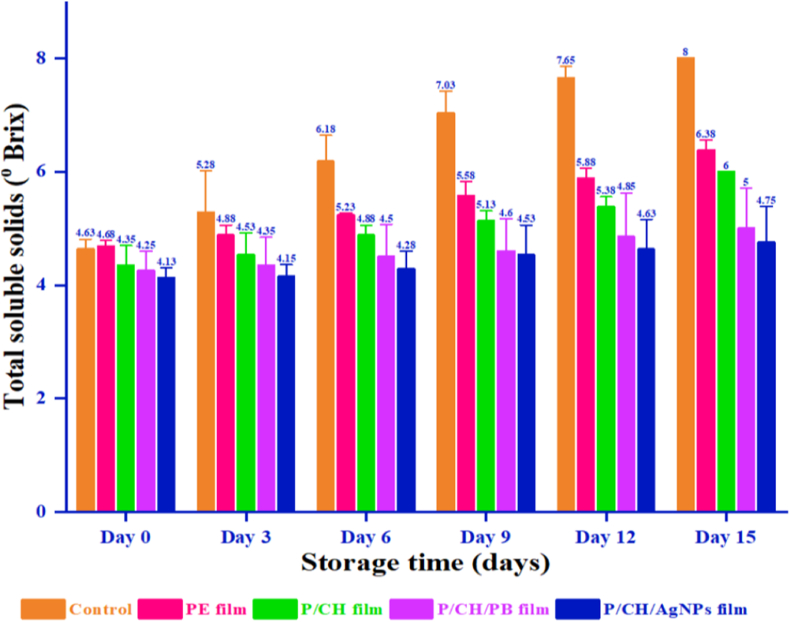
Concentration of total soluble solids in purple eggplant without film, coated with PE film, coated with P/CH film and coated P/CH/PB film during 15 days of storage at room temperature.

Total soluble solid (TSS) is an important parameter that was relatively stable during purple eggplant storage. Table 5 shows the total soluble solids in all samples during storage with control, PE film, P/CH film and P/CH/PB film. Under room conditions up to 3 days of storage, there was no considerable difference in changes in the total soluble solids of all samples, except of the control (Figure 8). From days 3–15 of storage, an increase in the content of soluble solids was observed all the samples, especially the control. This was probably related to the degradation of polysaccharides in purple eggplants during conservation due to water loss and an increase in sugar concentration. It was clear that the uncoated samples had the highest changes for the total soluble solids and the lowest changes were observed for the P/CH/PB film. Indeed, the amount of increase in the Brix percentage compared to initial values obtaining at the uncoated sample, the PE film, the P/CH film and the P/CH/PB film were 3.38%, 1.70%, 1.65%, and 1.00%, respectively. These results were in agreement with those reported by Weijie Lan et al. [76], that also showed an increase in total soluble solids in control and packaged mango during storage. Hereupon, using alternative food packaged with *Piper betle L.* leaf extract, could be considered as a benefit to avoid water loss by fruit transpiration of purple eggplant, and leading to increment of the vegetables shelf life.

**Table 5:** Visual aspect of purple eggplant without film, coated with PE film, coated with P/CH film and coated P/CH/PB film during 15 days of storage at room temperature.

Films	Storage time (days)					
	Day 1	Day 3	Day 6	Day 9	Day 12	Day 15
Uncoated						
PE film						
P/CH film						
P/CH/PB film

## 4 Conclusion

The pectin used in this research was prepared from passion fruit peel by acidic hot water extraction and the obtained pectin has a galacturonic acid content of 62.92%, degree of esterification of 50.74% and low molecular weight. The pectin/chitosan blend films and films incorporated with PB were prepared using the solution casting method. The chemical and physical structures of the prepared films were characterized by FT-IR, XRD, SEM analysis. Accordingly, the incorporation of PB extract into the P/CH films affected the mechanical properties and water vapor permeability of films as well as antibacterial activity. In addition, the P/CH/PB films showed excellent antibacterial activity and displayed positive results evaluating total soluble solids in purple eggplant during 15 days of storage. Therefore, the obtained P/CH/PB composite films have a potential to apply in active food packaging.

## Declarations

### Author contribution statement

Nguyen Thi Thanh Thuy: Conceived and designed the experiments; Performed the experiments; Analyzed and interpreted the data; Contributed reagents, materials, analysis tools or data; Wrote the paper.

Le Quoc Tu: Performed the experiments; Contributed reagents, materials, analysis tools or data.

Nguyen Thi Anh Tuyet: Conceived and designed the experiments; Analyzed and interpreted the data; Contributed reagents, materials, analysis tools or data.

Nguyen Thi My Lan: Analyzed and interpreted the data; Contributed reagents, materials, analysis tools or data.

Duyen Thi Cam Nguyen: Analyzed and interpreted the data; Contributed reagents, materials, analysis tools or data.

Thuan Van Tran: Analyzed and interpreted the data; Contributed reagents, materials, analysis tools or data; Wrote the paper.

### Funding statement

This work was supported by Nong Lam University Ho Chi Minh City, Vietnam under project ID: CS-CB22-KH-02.

### Data availability statement

Data included in article/supplementary material/referenced in article.

### Declaration of interest’s statement

The authors declare no conflict of interest.

### Additional information

No additional information is available for this paper.
